# Game-theoretical description of the go-or-grow dichotomy in tumor development for various settings and parameter constellations

**DOI:** 10.1038/s41598-023-43199-3

**Published:** 2023-10-05

**Authors:** Shalu Dwivedi, Christina Glock, Sebastian Germerodt, Heiko Stark, Stefan Schuster

**Affiliations:** 1 Department of Bioinformatics, Matthias Schleiden Institute, Friedrich Schiller University, Ernst-Abbe-Platz 2, 07743 Jena, Germany; 2 Institute of Zoology and Evolutionary Research, University of Jena, Erbertstr. 1, 07743 Jena, Germany

**Keywords:** Multicellular systems, Systems analysis, Cancer models, Metastasis, Applied mathematics

## Abstract

A medically important feature of several types of tumors is their ability to “decide” between staying at a primary site in the body or leaving it and forming metastases. The present theoretical study aims to provide a better understanding of the ultimate reasons for this so-called “go-or-grow” dichotomy. To that end, we use game theory, which has proven to be useful in analyzing the competition between tumors and healthy tissues or among different tumor cells. We begin by determining the game types in the Basanta–Hatzikirou–Deutsch model, depending on the parameter values. Thereafter, we suggest and analyze five modified variants of the model. For example, in the basic model, the deadlock game, Prisoner’s Dilemma, and hawk-dove game can occur. The modified versions lead to several additional game types, such as battle of the sexes, route-choice, and stag-hunt games. For some game types, all cells are predicted to stay on their original site (“grow phenotype”), while for other types, only a certain fraction stay and the other cells migrate away (“go phenotype”). If the nutrient supply at a distant site is high, all the cells are predicted to go. We discuss our predictions in terms of the pros and cons of caloric restriction and limitations of the supply of vitamins or methionine. Our results may help devise treatments to prevent metastasis.

## Introduction

### Motivation

A better understanding of the behavior and properties of tumors is important to improve therapies and reduce patient mortality. An important difference between benign and malignant (cancerous) tumors is that the former do not spread, whereas the latter do^1^. The spread of malignant tumors can occur either by invasion into neighboring tissues or by metastasis, that is, movement from an initial (primary) site to a distant (secondary) site within the body. Metastases usually drastically reduce the chance of healing^1,2^.

The alternatives of growing on the primary site or moving away can be considered as two different strategies in the sense of game theory. Understanding the ultimate reasons for the decision to use one of the two strategies is very helpful in tumor research. The dichotomy between the proliferation of a localized tumor (often, but not necessarily benign) and metastasis by cancer cells (malignant) is known as the go-or-grow dichotomy^3–5^.

### Game-theoretical background

The analysis of complex phenomena in cell and molecular biology is increasingly assisted by mathematical modeling and computational approaches. These approaches include evolutionary game theory^6–12^. This theory analyzes situations in which some cells, organisms, or populations (called agents or players) tend to optimize their properties to increase their fitness but interfere with each other, which may prevent them from attaining optimal states. The outcome for each player is quantified by a payoff and the equilibrium situation (the so-called Nash equilibrium or, in the case of many players in a population, an evolutionary stable strategy) can be determined^6,7,12,13^.

The general structure of payoff matrices for symmetric two-player two-strategy games is shown in Table 1:

**Table 1:** General structure of payoff matrices for symmetric two-player two-strategy games.

	Strategy 1	Strategy 2
Strategy 1	*R*, *R*	*S*, *T*
Strategy 2	*T*, *S*	*P*, *P*

*R* payoff for both players when choosing strategy 1, *P* payoff for both players when choosing strategy two, *S*, *T* payoffs when the two players choose different strategies^14^.

Symmetry means that both players have the same set of possible strategies and obtainable payoffs^6^. This is the case in the system under study because we consider competition among cancer cells (rather than, for example, among cancerous and healthy cells).

In total, there are four parameters. However, the number of relevant, independent parameters can be reduced to two. This is because for the classification of the game type, only the order relations among the parameters are relevant, and the addition of a constant to all payoffs and/or scaling by a positive factor does not change these relations. Moreover, we can renumber strategies to ensure that *R* > *P*. Renumbering strategies implies permuting both rows and columns. Thus, the only (exceptional) case not covered is *R* = *P*. Considering two variable parameters, Hauert^14^ classified the different types of symmetric 2-player 2-strategy games^12,15,16^.

Specific order relations characterize the 12 game types. For example, the Prisoner’s Dilemma is represented by:

1 $$T>R>P>S$$

and the hawk-dove game, by:

2 $$T>R>S>P$$

Hauert^14^ distinguished 12 generically different order relations, seven of which he assigned names. Later, names were assigned to all 12 types^12,15^.

From the results of symmetric two-player games, conclusions can be drawn regarding the frequency of strategies in populations^13^. For example, if the two-player game implies two pure Nash equilibria off the main diagonal, coexistence of the two strategies is observed in the population. If only one pure Nash equilibrium is found, virtually the entire population adopts the corresponding single strategy^17,18^. Some individuals may adopt the other strategy by chance, but will get a lower payoff. In general, stochastic fluctuations may occur around a Nash equilibrium.

### Application of game theory in tumor biology

A prominent example of the application of evolutionary game theory in cell biology is the description of the development and/or treatment of tumors^8–10,12,19–21^. The underlying idea is that tumor cells compete with healthy cells^8,9,22^, compete with each other^11,19^, or cooperate with each other^20,21^, thus showing features of Darwinian evolution^23^. The game character arises because the outcome for each cell depends not only on its own strategy but also on that of the other cells. Moreover, the “game” between the physician’s therapy and the cancer cells’ resistance strategies has been studied^24,25^.

Tumor cells or entire tumors can be considered as uncontrolled, replicative units that tend to maximize their fitness (e.g. growth rate). From the viewpoint of evolutionary biology, game theory is a useful tool in tumor biology^12,26,27^. Tumors can be considered as a regression in evolution because healthy cells of multicellular organisms usually cooperate with each other, whereas tumor cells show a more competitive behavior. For example, the Warburg effect, which implies that tumors mainly use glycolysis for ATP generation, can be explained by the maximization of the ATP production rate rather than yield^27,28^. As this pathway has a much lower ATP-over-glucose yield than respiration, resource utilization is less efficient.

Whether the emergence and development of tumors can be described by gradual changes or whether leaps (i.e., larger changes) should also be considered is an interesting question^29^. Observations have indicated that tumors can evolve in a punctuated, saltatory fashion. This is relevant to the game-theoretical description because it supports the consideration of distinct strategies.

Basanta et al.^11^ presented a study in which competition between tumor cells was considered as an evolutionary game. They defined two strategies, proliferative and motile. The former strategy corresponds to sessile cells in a localized tumor (often but not necessarily benign). The motile strategy means that the cell leaves the localized tumor (malignant; e.g. in the bloodstream), in search of more nutrients or space. This may or may not imply a metastasis. As “motile” usually refers to active movement^30^, while many metastatic cells are just moving passively in the bloodstream^31^, a better term may be “mobile”. However, here we stick with the terminology in the original model^11^ and use the term “motile”.

### Aims of the study

Here, we reanalyze the game suggested by Basanta, Hatzikirou and Deutsch^11^ (here called BHD model) in more detail and present five modified versions, considering possible alternative behaviors. The models under study here only require two or (in some extended games) three parameters: (1) the benefit *b* of being able to use the nutrients and space “alone”, that is, without competition, (2) the costs *c* of leaving the primary site and invading another tissue, and (3) a parameter *a* representing the accessibility of nutrients at the distant site. In the case of competition (e.g. if both players stay at the primary site), the benefit is *b*/2. For simplicity, we neglect the difference between the invasion of neighboring tissues and metastasis and combine both into the strategy of being motile.

We determine the types of games depending on parameter constellations. Different game types, depending on the parameter values also occur in other applications of game theory. For example, the sequence Harmony game, hawk-dove game, Prisoner’s Dilemma occurs in the secretion of extracellular enzymes by microorganisms^17,18^.

A type of tumor that may or may not form metastases is the hepatocellular carcinoma^32^. Hence, this is a suitable representative example of a tumor showing both phenotypes (go or grow). The most frequent sites of metastasis from this carcinoma are the lungs, bones, and abdominal lymph nodes.

Tumors consist of more than one cell. The model can also be applied to cases in which tumor cell aggregates rather than particular cells compete with each other^31^. However, this would require that these aggregates are of approximately the same size, so that they can be described by the same parameters.

Moreover, we will interpret the results of the game-theoretical analysis in terms of tumor biology and oncology. In particular, we will deal with the question of which parameters, for example, concerning the restriction of calorie and vitamin supply, should be increased or decreased to suppress metastasis.

## The Basanta–Hatzikirou-Deutsch model and analysis in terms of game types

**Figure 1:**
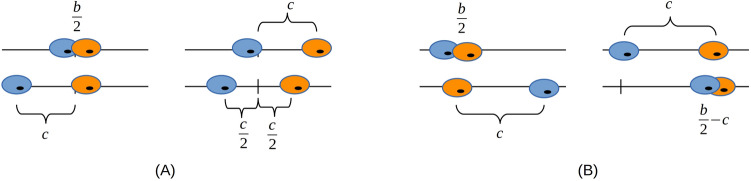
Graphical representation of the game in which the motile cells (or cell aggregates) move in the (**A**) opposite direction and (**B**) same direction.* b*, availability of nutrients; *c*, costs for motility. (**A**) The four panels correspond to the four boxes in the payoff matrices of the BHD model and Modification I (Table 2). Two sessile cells have to share the nutrients so that each of them obtains *b*/2, while both of them obtain the full amount if one of them moves away (which is *b* in the BHD model and another parameter, *a*, in Modification I). It is assumed that when both cells move away, only half of the costs are needed in comparison to when one cell moves away because they need to go only about half the distance each to find more nutrients. In that case, both cells again have access to the full amount of nutrients. (**B**) The four panels correspond to the boxes in the payoff matrix of Modification IV (Table 2).

The BHD model^11^ describing the interaction between tumor cells (see Introduction) involves two parameters: the availability of nutrients, *b*, and the cost of motility, *c* (Fig. 1A). Its authors suggested the payoff matrix shown in Table 2 (box corresponding to BHD model).

**Table 2:** Payoff matrices for the Basanta–Hatzikirou–Deutsch (BHD) model^11^ and five modifications.

	Proliferative	Motile
BHD model		
Proliferative	$\frac{b}{2},\frac{b}{2}$	$b,b-c$
Motile	$b-c,b$	$b-\frac{c}{2},b-\frac{c}{2}$
Modification I		
Proliferative	$\frac{b}{2},\frac{b}{2}$	$b,a-c$
Motile	$a-c,b$	$a-\frac{c}{2},a-\frac{c}{2}$
Modification II		
Proliferative	$\frac{b}{2},\frac{b}{2}$	$b,b-c$
Motile	$b-c,b$	$b-c,b-c$
Modification III		
Proliferative	$\frac{b}{2},\frac{b}{2}$	$b,a-c$
Motile	$a-c,b$	$a-c,a-c$
Modification IV		
Proliferative	$\frac{b}{2},\frac{b}{2}$	$b,b-c$
Motile	$b-c,b$	$\frac{b}{2}-c,\frac{b}{2}-c$
Modification V		
Proliferative	$\frac{b}{2},\frac{b}{2}$	$b,a-c$
Motile	$a-c,b$	$\frac{a}{2}-c,\frac{a}{2}-c$

*Modification I* Directions of possible movement are opposite to each other and the amount of nutrients depends on the site, *Modification II* Directions of possible movement are opposite to each other, the same amount of nutrients is available at every place and the cost is independent of the covered distance. *Modification III* Directions of possible movement are opposite to each other, the amount of nutrients depends on the site and the cost is independent of the distance covered. *Modification IV* Direction of possible movement is the same for the two cells, the amount of nutrients is constant at every location and the cost is independent of the covered distance. *Modification V* Direction of possible movement is the same for the two cells, the amount of nutrients depends on the site and the cost is independent of the distance covered.

**Figure 2:**
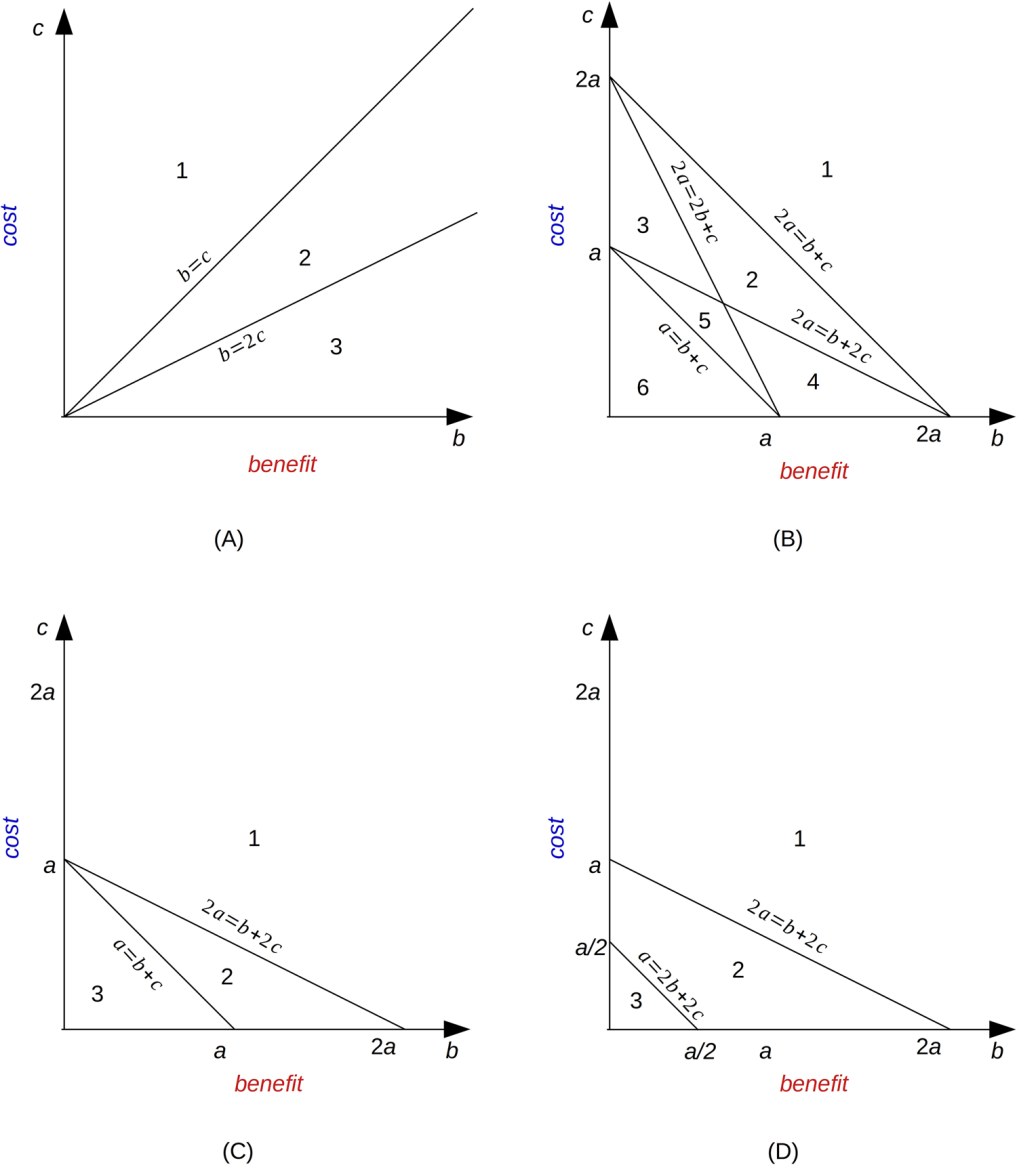
Benefit–cost plane for the BHD model and five modifications. (**A**) Three areas corresponding to the three game types for the BHD model and Modifications II and IV. BHD model: 1, deadlock game; 2, Prisoner’s Dilemma; 3, hawk-dove game. Modification II: Joint regions 1 and 2, route-choice/deadlock; 3, hawk-dove/leader. Modification IV: Joint regions 1 and 2, route-choice; 3, battle of the sexes. (**B**) Six areas corresponding to the six game types for Modification I. Regions: 1, deadlock game; 2, Prisoner’s Dilemma; 3, stag-hunt; 4, hawk-dove game; 5, harmony I; 6, harmony II. In regions 2–6, the rows and also columns in the payoff matrix are swapped. (**C**) Three areas corresponding to the three game types for Modification III. 1, route-choice/deadlock; 2, hawk-dove/leader; 3, route-choice/harmony II. In regions 2 and 3, the rows and also columns in the payoff matrix are swapped. (**D**) Three areas corresponding to the game types for Modification V. Regions 1 and 3, route-choice game; 2, battle of the sexes. In regions 2 and 3, the rows and also columns in the payoff matrix are swapped. For the Nash equilibria of the various games, see text.

Now, we want to determine the type of game this matrix (Table 2, BHD model) corresponds to, depending on the parameter values. Note that in the payoff matrices shown, the inequality *R* > *P* is not always immediately fulfilled. If not, then we permute both rows and columns. Depending on the order relationship between benefits and costs, we can distinguish three different cases (Fig. 2A).

### Case (1): low benefit-to-cost ratio: Deadlock game

In the above payoff matrix (Table 2, BHD model), the order relation *R* > *P* holds if $\frac{b}{2}>b-\frac{c}{2}$, which is equivalent to *c* > *b*. Under this condition, we do not need permute rows or columns. Moreover, this implies that:

3 $$b>\frac{b}{2}>b-\frac{c}{2}>b-c$$

With the general notation for payoffs, this reads:

4 $$S>R>P>T$$

This is the order relation of the deadlock game^12,14^.

The name “deadlock game” comes from a cover story in which two car drivers can choose between a highway and a narrow road^12^. The best case is driving on the highway alone (payoff *S*). Thus, a driver opting for the narrow road provides a major advantage to the other driver. Similarly, a motile tumor cell provides a high advantage to the other cell, which can remain stationary and then has the highest payoff. Sharing the highway provides the second-best payoff (*R*), and sharing the narrow road (payoff *P*) is better than using it alone (*T*). The latter order relation may be explained in that drivers can help each other in the case of an accident.

The Nash equilibrium is that they both stay on the highway (upper-left cell of the matrix). Therefore, none of the drivers receives the highest payoff; hence, the name deadlock highlights a dilemma for both players. In terms of the metastasis game, the Nash equilibrium is “proliferative / proliferative”, implying that both cells stay.

### Case (2): intermediate benefit-to-cost ratio: Prisoner’s dilemma

Now we consider the case 2*c* > *b* > *c*. Then, we permute rows and columns so that again *R* > *P*. In this case, the payoffs fulfil the order relation:

5 $$b>b-\frac{c}{2}>\frac{b}{2}>b-c$$

that is,

6 $$T>R>P>S$$

which corresponds to the Prisoner’s Dilemma. For a cover story of the Prisoner’s Dilemma, see^7,33^. This game type has been discussed in the context of cancer earlier^20,22^, and in many other systems studied in cell biology^12,17,18^. It has a single, symmetric Nash equilibrium, which reads, for the tumor cells, “proliferative/proliferative”.

Thus, the deadlock game and the Prisoner’s Dilemma lead to the same outcome in the tumor game,. The difference is that, in the former game, only one cell could have a higher payoff in a situation different from the Nash equilibrium, while paradoxically, both cells could be better off in the Prisoner’s Dilemma, notably in the other symmetric situation (which here reads “motile/motile”). However, it is not stable because there is a temptation to switch to the defective strategy (here “proliferative”) which would provide the highest payoff. Because both players are tempted, both of them get stuck in a suboptimal situation.

### Case (3): high benefit-to-cost ratio: Hawk-dove game

If *b* > 2*c*, we can again use the permuted form of the payoff matrix, as given in Table 2, the BHD model. The payoffs now fulfil the following order relation:

7 $$b>b-\frac{c}{2}>b-c>\frac{b}{2}$$

that is,

8 $$T>R>S > P$$

This corresponds to the hawk-dove game, also known as snowdrift game or game of chicken [Eq. (2)], which has two asymmetric Nash equilibria. For the cover story of the hawk-dove game, see^7,33^. This game type has been discussed in the context of cancer earlier^20,22^ and many other systems in cell biology^12,17,18^.

For the game under study, the Nash equilibria read “proliferative/motile” and “motile/proliferative”. That is, one of the two cells (the “dove”) migrates away, while the other (the “hawk”) stays and benefits from the highest payoff. For the former cell, it is better in terms of payoffs to be motile than to try to stay as well. Which of the two cells becomes the “dove” depends on initial fluctuations or small differences among the cells (see “Summary in terms of game types” below).

### Role of information

It is worth noting the role of information. In complete-information games, players have all the knowledge about the game (players, strategies and payoffs) but they may not observe the actions of their counterparts. In perfect information games, each player has all the knowledge of the other players and their previous actions^33^. Players might have perfect but incomplete information; for example, when the moves of the other players, but not their resulting payoffs are known. Interestingly, there is experimental evidence that primary tumors and distant site metastases communicate in both directions^34,35^.

The BHD model^11^ was discussed by the authors in view of the population. Thus, the exchange of information among players is less important. It is determined by mutation (or epigenetic regulation) and selection whether a player is successful. The player does not need to know the consequences of choosing a certain strategy but will just experience them.

Assume that a certain fraction *p* of a population of cells or cell aggregates opts for one strategy and a fraction 1 − *p* for the other. If the interactions within the population are considered as a series of two-player encounters, it can be shown that the equilibrium fraction *p* equals the probability of choosing the first strategy in a mixed Nash equilibrium provided that 0 < *p* < 1^13^.

Games leading to just one pure Nash equilibrium, such as the deadlock game and Prisoner’s Dilemma, do not have a mixed Nash equilibrium. In the case of the tumor game under study, all cells adopt the same strategy, such that *p* = 0 or *p* = 1. In games leading to two pure Nash equilibria, a mixed Nash equilibrium occurs that is adopted by the population (at least in theory). For this, fraction *p* can be calculated from the four payoffs *R*, *S*, *T,* and *P*^6,11,13^:

9 $$p=\frac{P-S}{R-T-S+P}$$

In cases where this formula gives *p* < 0 or *p* > 1, only one pure and no mixed Nash equilibrium occurs.

## Modifications of the model in terms of benefits and costs

### I: Different supply of nutrients

In the BHD model, it is assumed that the total amount of nutrients (e.g. glucose, glutamine, or possibly oxygen) is the same everywhere, and cancer cells can either use it alone or share it. However, the amount of nutrients usually depends on their location in the body. Therefore, a new parameter *a* is introduced here that represents nutrient availability at a distant site (Table 2, Modification I).

Three parameters must be considered: *a*, *b* and *c*. This leads to six game types, that differ in order relations among payoffs (Fig. 2B and Table 3).

**Table 3:** Game analysis for Modification I (see payoff matrix in Table 2).

Case	Order relation of payoffs	Game type	Nash equilibrium
(1) 2*a* < *b* + *c*	*S* > *R* > *P* > *T*	Deadlock	(Proliferative, proliferative)
(2) 2*a* > *b* + *c*, 2*a* < 2*b* + *c*, 2*a* < *b* + 2*c*	*T* > *R* > *P* > *S*	Prisoner’s Dilemma	(Proliferative, proliferative)
(3) 2*a* > 2*b* + *c*, 2*a* < *b* + 2*c*	*R* > *T* > *P* > *S*	Stag-hunt	(Motile, motile), (proliferative, proliferative)
(4) 2*a* < 2*b* + *c*, 2*a* > *b* + 2*c*	*T* > *R* > *S* > *P*	Hawk-dove	(Proliferative, motile), (motile, proliferative)
(5) 2*a* > 2*b* + *c*, 2*a* > *b* + 2*c*, * a* < *b* + *c*	*R* > *T* > *S* > *P*	Harmony I	(Motile, motile)
(6) *a* > *b* + *c*	*R* > *S* > *T* > *P*	Harmony II	(Motile, motile)

In case 1, strategy 1 = proliferative, whereas in cases 2–6, strategies are renumbered so that strategy 1 = motile.

The game “stag-hunt” comes from a cover story in which two huntsmen have the choice to hunt a stag or two hares and can be successful in catching the stag only if they team up^21,36^, similarly to the story ‘Lions and Antelope’^7^. If they go together for a stag, they must share it. If they hunt hares, they can keep one each to themselves, but half of a stag is worth more than a hare. This game has two pure Nash equilibria on the main diagonal of the payoff matrix. For the situation of tumor cells, these equilibria read “motile/motile” and “proliferative/proliferative” (Table 2, Modification I, permuted). Both cells either choose the motile strategy and move away to a new place or proliferate, staying at the initial place and sharinge nutrients. Since *R* > *P*, it may be assumed that rational players would opt for the cooperative hunting strategy. However, cancer cells do not have cognitive capabilities and, thus, may get stuck in the other Nash equilibrium, which is “proliferative/proliferative”.

The name harmony game comes from the property of its payoff matrix, in which both players obtain the maximum payoff in the (then unique) Nash equilibrium (a situation of harmony). For the tumor situation, this implies that both cells receive the highest payoffs when they move away to a new site because the availability of nutrients minus travel cost is worth more than the nutrients at the initial place. Hence, the Nash equilibrium is “motile/motile” (Table 2, Modification I, permuted). The games “Harmony I” and “Harmony II” are very similar to each other. The only difference is that if a cell deviates from the cooperative strategy in Harmony I, it reduces its payoff to a lesser extent than the payoff of the other player (*T* > *S*), while it is the other way round in Harmony II (*S* > *T*). This has no effect on the Nash equilibrium.

To prevent the tumor from moving, the conditions (parameters) of the tumor environment should lead only to the “proliferative/proliferative” Nash equilibrium. Therefore, cases 1 + 2 are the desired states, that is, *2a* < *2b* + *c and 2a* < *b* + *2c*. This may be achieved by increasing *b* (local nutrients of the tumor) or decreasing *a* (nutrition at other sites). It may be more difficult to increase *c* (the cost of moving), see “Discussion”.

### II: Same cost for both tumor cells when moving

In the basic model, it is assumed that when both cells move away, only half of the costs are required in comparison to when one cell moves away (Table 2, BHD model). However, a plausible assumption ist that moving away always implies the same cost. This new assumption is now considered in the payoff matrix (see Table 2, Modification II). Depending on the order relationship between benefits and costs, we can distinguish two different cases (Fig. 2A and Table 4).

**Table 4:** Game analysis for Modification II (see payoff matrix in Table 2).

Case	Order relation of payoffs	Game type	Nash equilibrium
(1) *b* < 2*c*	*S* > *R* > *T* = *P*	Route-choice/deadlock	(Proliferative, proliferative)
(2)* b* > 2*c*	*T* > *S* = *R* > *P*	Hawk-dove/leader	(Proliferative, motile) (motile, proliferative)

Slashes indicate that the game is on the boundary between the two given game types. In case 2, the strategies are renumbered so that strategy 1 is motile.

Here, we need not distinguish between low- and intermediate-benefit cases because they lead to the same game type, notably one on the boundary between the route-choice and deadlock games, whereas the high-benefit case is in between the hawk-dove and leader games (Table 4). The model corresponds to the boundary between the two game types because two payoffs are equal to each other (*b*–*c*).

The cover story of the route-choice game (also known as deadlock II)^12,37^ is similar to that of the deadlock game. The only difference is that driving alone on a narrow road is better than sharing it.

The cover story of the leader game comes from a strategic game between two firms. It is related to the Stackelberg game^7,25^, which may be asymmetric. It is named after German economist Heinrich Freiherr von Stackelberg who published an early monograph on economics^38^. One of the companies, the leader firm, moves first and, thus, always has an advantage. The leader game has two pure Nash equilibria off the main diagonal in the payoff matrix^62^, similar to the hawk–dove game. For the system under study, the Nash equilibria are shown in Table 4. One cell migrates away (corresponding to ‘the follower firm’, although that term from the cover story may be misleading here) and the other cell can stay (‘leader firm’).

### III: Same cost and different supply of nutrients

For a combination of Modifications I and II, a parameter *a* is used that represents the different availability of nutrition at different locations. This provides a new payoff matrix (Table 2, Modification III). Depending on the order relation between the benefit and cost, we can combine the six cases from Modification I into three different cases (Fig. 2C and Table 5).

**Table 5:** Game analysis for Modification III (see payoff matrix in Table 2).

Case	Order relation of payoffs	Game type	Nash equilibrium
(1) 2*a* < *b* + 2*c*	*S* > *R* > *P* = *T*	Route-choice/deadlock	(Proliferative, proliferative)
(2) 2*a* > *b* + 2*c*, *a* < *b* + *c*	*T* > *S* = *R* > *P*	Hawk-dove/leader	(Proliferative, motile) (motile, proliferative)
(3) *a* > *b* + *c*	*S* = *R* > *T* > *P*	Route-choice/Harmony II	(Proliferative, proliferative) (motile, motile)

Slashes indicate that the game is on the boundary between the two given game types. In cases 2 and 3, the strategies are renumbered such that strategy 1 is motile.

### IV: Site-independent nutrient supply, also motile cells have to share nutrients

Here, we consider at scenario in which tumor cells move in the same direction, the availability of nutrients is the same at both positions, and the cost for motility is always the same (Fig. 1B). Indeed, it has been observed that some tumor cells move in the same direction as the so-called stream^30^.

The payoff matrix for this model modification is presented in Table 2 (Modification IV). There are two different cases based on the two parameters *b* and *c*. The order relation *R* > *P* holds in every case, that is, $b/2>b/2-c$. Therefore, we do not need to permute the rows or columns. Depending on the order relationship between benefits and costs, we can distinguish two different cases (Fig. 2A and Table 6).

**Table 6:** Game analysis for Modification IV (see payoff matrix in Table 2).

Case	Order relation of payoffs	Game type	Nash Equilibrium
(1) *b* < 2*c*	*S* > *R* > *T* > *P*	Route-choice	(Proliferative, proliferative)
(2) *b* > 2*c*	*S* > *T* > *R* > *P*	Battle of the sexes	(Proliferative, motile) (motile, proliferative)

Case 1 (low/intermediate benefit) corresponds to the route-choice game, whereas the high-benefit case corresponds to the battle of the sexes game (Table 6). The latter game has a cover story in which a couple wishes to go out together^7,36^. To be together, rather than being in different places is their highest priority. However, the husband and wife have different preferences: watching football games and going to the opera, respectively. By considering the strategies ‘my preference’ and ‘the other’s preference’, the game can be written as a symmetric one. The game has two pure Nash equilibria: one player chooses ‘my preference’ and the other one selects ‘the other’s preference’. Thus, in the payoff matrix, the two Nash equilibria are situated off the main diagonal, as in the hawk-dove and leader games. Here, the game leads to the “proliferative/motile” and “motile/proliferative” Nash equilibria (Table 2, Modification IV). That is, one cell migrates away (corresponding to ‘the other’s preference’) and the other cell can stay (‘my preference’).

It might be difficult to recognize the analogy between the cover story of the battle of the sexes game with the situation under study. It is worth noting that the analogy is purely formal rather than related to an effect of the gender of cancer patients. While in either Nash equilibrium of that game, the two partners go to the same place, the two tumor cells end up at different places. This is because the strategies are renamed ‘my preference’ and ‘the other’s preference’, so that the two partners use different strategies in the Nash equilibrium. A further analogy is that the cell opting to go away provides an even larger advantage to the other cell than to itself, just as the partner opting for ‘the other’s preference’.

### V: Site-dependent nutrient supply, also motile cells have to share nutrients

Now, we consider the scenario where tumor cells move in the same direction, but the availability of nutrients is different at both positions, that is, ‘*a*’ and ‘*b*’, and the cost for motility is ‘*c*’ (Table 2, Modification V). According to the game types, there are three cases dependent on the three parameters (Fig. 2D and Table 7). In Table 2 (Modification V), inequalities $b>b/2$ and $a-c>a/2-c$ always hold, which implies that the order relations *S* > *R* and *T* > *P* are always true for every case.

**Table 7:** Game analysis for Modification V (see payoff matrix in Table 2).

Case	Order relation of payoffs	Game type	Nash equilibrium
(1) 2*a* < *b* + 2*c*	*S* > *R* > *T* > *P*	Route-choice	(Proliferative, proliferative)
(2) 2*a* > *b* + 2*c,* *a* < 2*b* + 2*c*	*S* > *T* > *R* > *P*	Battle of the sexes	(Proliferative, motile) (motile, proliferative)
(3) *a* > 2*b* + 2*c*	*S* > *R* > *T* > *P*	Route-choice	(Proliferative, proliferative)

In case 1, the proliferative strategy is number 1, and in cases 2 and 3, number 2.

Interestingly, cases (1) and (3) have the same classification of the game, although they are not connected in the benefit–cost plane, notably ‘route-choice’. Note that these cases differ in the order of rows and columns of the payoff matrix. The remaining case corresponds to the ‘battle of the sexes’.

### Summary in terms of game types

**Figure 3:**
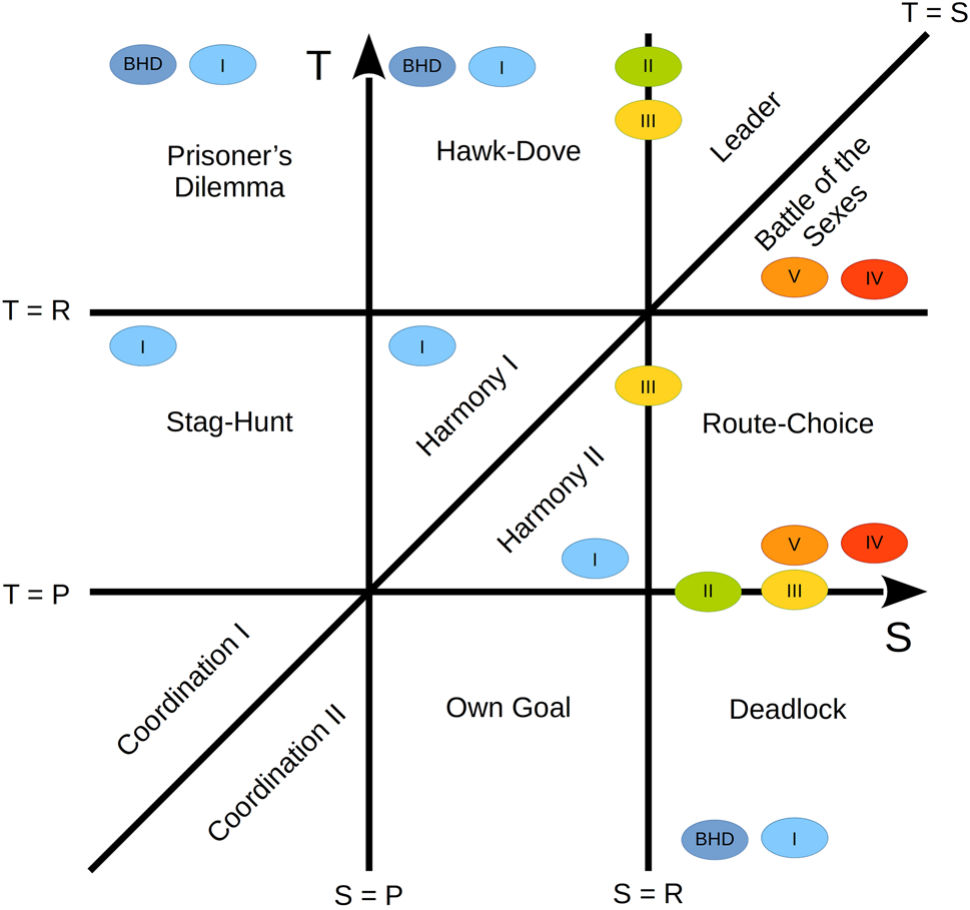
Classification diagram of the games in the *S*, *T* plane. The four parameters in symmetric two-player two-strategy games can be reduced to two because the addition of a constant to all payoffs and/or scaling by a positive factor does not change the order relations among payoffs. The possible game types for the BHD and the five model modifications (indicated by Roman numbers) analyzed here are shown. The location of the ellipses in the diagram represents the order relations among *R*, *S*, *T*, and *P*. Ellipses on a boundary represent games in which an order relation among payoffs is an equality, so that they belong to two game types.

Figure 3 shows, in the plane spanned by the two payoffs *S* and *T*, all game types and in which modifications of the BHD model they occur. If the game type changes upon a gradual change of *S* or *T*, these types should correspond to adjacent regions in Fig. 3, unless rows (and also columns) need to be permuted because the order relation between *R* and *P* is changed.

The Prisoner’s Dilemma is relevant in the tumor games under study if the cost has an intermediate value so that it would be favorable for the two cells if they both moved, while an even higher incentive is to stay while the other cell moves. It would pay if both cells go simultaneously, but that would require coordination among them.

In the hawk-dove game, as applied to tumors, the hawk remains at the initial site and thus obtains a higher payoff than the dove, which moves away. Nevertheless, the dove is better off than if it stayed because if both remained at the site, they would have to share the nutrients. The decision which of the two cells “surrenders” and goes away may depend on slight physiological differences between them or between the blood vessels in which they may move, or on initial fluctuations. Such fluctuations show a self-amplifying effect; once one of the cells starts moving, the incentive of the other cell to stay becomes even stronger. This, in turn, strengthens the incentive for the former cell to move away.

The leader game is related to the hawk-dove game, in which the two cells use different strategies in the Nash equilibrium. The difference is that the go type obtains a higher payoff if the other cell stays than if both cells go. Also in battle of the sexes, the two cells use different strategies in the Nash equilibrium. One cell follows the ‘my preference’ strategy and the other one, the ‘other’s preference’ strategy. One of the cells spends the cost for motility to achieve a better payoff.

In the stag-hunt game, both cells either stay at the initial site or move away to obtain a better payoff. In a harmony game, both cells can maximize their payoffs by cooperating with each other. Because, in that case, the availability of nutrients at distant sites is higher than at the initial site, even if the costs are taken into account, both cells will receive more payoff if they move away instead of staying. Thus, both cells choose the motile strategy.

Since, in the route-choice game, both cells would go in the same direction in the corresponding model, it is better to stay at the initial site than to move to another site and share nutrients there again and, in addition, bear the cost of motility. In the deadlock game, it is better for cells to stay and proliferate.

### Ethics compliance

The article does not contain any studies involving human or animal participants.

## Discussion

Here, we analyzed a model for competition among tumor cells originally proposed by Basanta et al.^11^. We have suggested five modifications of the basic model and analyzed them as well, with respect to their payoff matrices (Table 2), game types and Nash equilibria (Fig. 3 and Tables 3, 4, 5, 6, 7). All of these model variants represent symmetric two-player, two-strategy games. They are described by the two parameters *b* (benefit at the initial site) and *c* (cost for motility), while in Modifications I, III, and V, also the parameter *a* (benefit at distant site) is used.

As the cells’ decision is assumed to depend on (among other parameters) the nutrient availability at distant sites, the question arises as to how the cells at the primary site obtain this information. It has becomes increasingly clear that cancer cells communicate with each other and their environment, notably over longer distances than previously realized^34,35,39^. Moreover, an interpretation in terms of trial-and-error can be put forward: some cells start moving and form pre-metastatic niches (PMNs). Only those cells that find a supportive tissue microenvironment will succeed in metastatic colonization^40^.

Our results are of interest in view of the go-or-grow behavior^3–5^. We found that, depending on the parameter values, the following game types can occur in the basic model: deadlock game, Prisoner’s Dilemma, and hawk-dove game (Fig. 3). For the former two game types, the model predicts that no metastatic cancer cells (go type) are formed, and that the tumor cells remain sessile (grow type). The grow type could be malignant or benign (also possibly dangerous), whereas the go type is always malignant.

Leukemia is not directly covered because this type of tumor cells is always mobile. However, in the broader sense, we can describe it by the games considered here if staying in the blood is identified with the grow type and invading other organs with the go type.

### Prospects for application

For simplicity, we neglected many features of tumors, such as their ability to shape their environment, for example, by angiogenesis. Nevertheless, this theoretical study may be helpful in providing guidelines or suggestions for improving the medical treatment of tumors. Since metastases are usually more dangerous than localized tumors, one may try to change the parameters by intervention in such a way that the symmetric Nash equilibrium corresponding to localized tumors is attained, as long as it is impossible to kill or remove the tumor. When ‘treating to kill’ does not work, it may be feasible to use therapies to ‘treat to contain’^24^.

It would certainly be of interest to validate these results in experiments. Our model provides testable predictions. The first option for therapeutic intervention is nutrient limitation, that is, lowering *b* (or possibly *a* in addition), which should favor the go-type. This parameter can be adjusted according to the patient’s diet^11,41–43^.

Glucose and glutamine are the main sources of carbon and energy in tumor cells. The Warburg effect is well known for glucose^10,28,41,44^. Thus, a large amount of glucose is required. Glutamine is not only a major source of energy but also a nitrogen source, especially for cells of the hematopoietic or myeloid lineages^42,45^. Importantly, myeloid cells are considered the origin of many metastatic cancers^43^. Thus, restriction of glucose and/or glutamine supply, possibly combined with drugs targeting glutamine metabolism, appears to be a promising anti-tumor strategy.

Methionine is another important amino acid^46,47^. It was demonstrated with tumors in rats that a methionine-poor diet slowed down tumor growth considerably^48^. It could also be shown with positron emission tomography imaging^49^ that cancer and normal cells may differ more in their requirement for methionine than for glucose^47^.

However, nutrient-restriction methods are targeted for long-term treatment and are, more relevant for the prevention of future tumors^43^. It is well known that the Western diet is associated with an increased incidence of many cancers such as prostate, breast, and colorectal cancer^50,51^. In general, however, it is important to consider the patient's nutritional status, stage of therapy, and recommendations of the oncologist and dietitian. This is because cachexia (muscle loss) can result from this treatment and has been linked to changes in the immune system and metabolism^51^. Instead of simple calorie restriction, intermittent fasting, calorie-restriction mimetic drugs, or ketogenic diets are worth testing^51^. More work is needed in the future, both in mathematical modeling and experiment, to analyze the complex interplay of metabolism and cancer and to determine the optimal nutritional regime for cancer patients^52^.

In this context, the supplementation of vitamins in the diet of patients with cancer is worth discussing. It is called a double-edged sword^53^ and has several pros and cons. For example, thiamine (vitamin B1) is often deficient in patients with advanced cancer and is, then, supplemented as nutritional support^53,54^. However, thiamine pyrophosphate is the cofactor of transketolase and is thus, required for cell proliferation because it is involved in ribose synthesis. Accordingly, Metabolic Control Analysis showed that thiamine administration significantly increased tumor growth^54^. Generally, vitamins promote health. In contrast, the pentose phosphate pathway, which is usually upregulated in tumors, is supported. However, limiting the thiamine supply might cause a shift from “grow” to “go” and thus promote metastases, possibly depending on the tissue. This adds a point to the pro side. It should be noted that strong proliferation of localized tumors can also be dangerous.

An option worth considering is to change nutrient utilization rather than nutrient availability in cancer therapy. For example, oxidative phosphorylation may be induced by specific drugs to reduce the Warburg effect^44^. This may lower the incentive for tumor cells to move to distant sites.

Second, besides nutrient availability (parameters *a* and *b*), the costs (parameter c) are also subject to medical intervention changes. It might be possible to design therapies that impede the detachment of tumor cells from the extra-cellular matrix by downregulating integrins or other structural proteins^11^. In fact, integrin function-blocking monoclonal antibodies were shown to inhibit tumor cell migration^55^, which can be interpreted as an increase in costs. Moreover, blocking receptors on the surface of metastatic cells hinders the invasion of other tissues^39^ and regulators of structural proteins are potential therapeutic targets^30^.

A third way of therapeutic intervention is to block the preparation of PMNs, which includes lowering glucose availability in PMNs and preventing an increase in vascular permeability^40^. This is likely to affect nutrient availability (*b*) and the costs (*c*), respectively.

### Outlook

In this paper, the processes within an individual host have been studied. However, infections of or by other individuals are worth being studied as well^56^. This would be the case, for example, with sticker tumors in dogs, devil facial tumor disease in Tasmanian devils^57^ and microchimerism in humans, that is the transfer of cells between fetus and pregnant woman^58^.

This study is also relevant to many other situations in ecology, notably when two individuals/groups of the same species or two competing species can choose between two different habitats. The decision is to share one habitat with another species or migrate to another habitat and invest the cost in that move. This has been previously studied for spider colonies^59^. The effect of food availability on bee hives has been modeled^60^. It is interesting, in future studies, to compare models of cancer development with models in animal ecology.

A further interesting extension is to study migration on graphs (e.g. lattices) rather than just between one primary and one secondary site and to introduce co-evolutionary rules saying how players are behaving and are rewarded when becoming neighbors on the graph^61^. Such rules could take into account properties of cells such as their lineage and age. The graph may be given a priori or may result, as a random graph, from a continuous movement of cells in space^62^.

## Data Availability

All data generated or analyzed during this study are included in this published article. All research data supporting this publication are included in the paper.
